# Protein–Protein Interaction Network Extraction Using Text Mining Methods Adds Insight into Autism Spectrum Disorder

**DOI:** 10.3390/biology12101344

**Published:** 2023-10-18

**Authors:** Leena Nezamuldeen, Mohsin Saleet Jafri

**Affiliations:** 1 School of Systems Biology, George Mason University, Fairfax, VA 22030, USA; 2 King Fahd Medical Research Centre, King Abdulaziz University, Jeddah 21589, Saudi Arabia; lnnezamuldeen@kau.edu.sa; 3 Center for Biomedical Engineering and Technology, University of Maryland School of Medicine, Baltimore, MD 21201, USA

**Keywords:** artificial intelligence, PPI, protein–protein interaction, text mining, BiLSTM, recurrent neural network

## Abstract

**Simple Summary:**

Research on proteins and their interactions with other proteins yields many new findings that help explain how diseases emerge. However, manual curation of scientific literature delays new discoveries in the field. Artificial intelligence and deep learning techniques have played a significant part in information extraction from textual forms. In this study, we used text mining and artificial intelligence techniques to address the issue of extracting protein–protein interaction networks from the vast amount of scientific research literature. We have created an automated system consisting of three models using deep learning and natural language processing methods. The accuracy of our first model, which employs recurrent neural networks using sentiment analysis, was 95%. Additionally, the accuracy of our second model, which employs the named entity recognition technique in NLP, was effective and achieved an accuracy of 98%. In comparison to the protein interaction network, we discovered by manual curation of more than 30 articles on Autism Spectrum Disorder, that the automated system testing on 6027 abstracts was successful in developing the network of interactions and provided an improved view. Discovering these networks will greatly help physicians and scientists understand how these molecules interact for physiological, pharmacological, and pathological insight.

**Abstract:**

Text mining methods are being developed to assimilate the volume of biomedical textual materials that are continually expanding. Understanding protein–protein interaction (PPI) deficits would assist in explaining the genesis of diseases. In this study, we designed an automated system to extract PPIs from the biomedical literature that uses a deep learning sentence classification model, a pretrained word embedding, and a BiLSTM recurrent neural network with additional layers, a conditional random field (CRF) named entity recognition (NER) model, and shortest-dependency path (SDP) model using the SpaCy library in Python. The automated system ensures that it targets sentences that contain PPIs and not just these proteins mentioned in the framework of disease discovery or other context. Our first model achieved 13% greater precision on the Aimed/BioInfr benchmark corpus than the previous state-of-the-art BiLSTM neural network models. The NER model presented in this study achieved 98% precision on the Aimed/BioInfr corpus over previous models. In order to facilitate the production of an accurate representation of the PPI network, the processes were developed to systematically map the protein interactions in the texts. Overall, evaluating our system through the use of 6027 abstracts pertaining to seven proteins associated with Autism Spectrum Disorder completed the manually curated PPI network for these proteins. When it comes to complicated diseases, these networks would assist in understanding how PPI deficits contribute to disease development while also emphasizing the influence of interactions on protein function and biological processes.

## 1. Introduction

Intracellular and extracellular proteins are the core building blocks of many intracellular signaling pathways [1]. Protein–protein interactions (PPIs) describe the primary pathways for cell function and have been associated with disease development. With reference to the available PPI databases, such as BioPAX [2], Pathway Commons [3], and CausalPath [4], discovering the most recent form of interactions between proteins and other elements remains difficult [5]. With the tremendous expansion of biomedical literature, manualcuration of this literature creates difficulties in having revised PPI figures and revealing the hidden knowledge contained within the unstructured text. Protein–protein interaction prediction online applications that use text mining approaches are GENEMANIA [6] and STRING DB [7]. Their method for predicting PPI networks is unique and incorporates seven evidence-based channels, namely neighborhood, fusion, co-occurrence, co-expression, experiments, database, and text mining model, trained on biomedical RoBERTa-large model, with different sub-scores provided from each channel. These channels detect the PPI from different sources related to biological information, including genomic databases, functional genomic databases, protein databases, pathway databases, and the PubMed database. Their method of operation is as follows: If you submit the name of a protein, the system will predict the proteins with which it interacts based on their functional partners. If you enter more than one protein, however, the PPI network edges will indicate where these two proteins were mentioned, and the user will need to return to the publication to determine the relationship between the two proteins, if there is one, or if they were mentioned as biomarkers for a disease (for example [8]).

With the emergence of Natural Language Processing (NLP) methods, PPI extraction from biomedical abstracts becomes feasible. The most challenging aspect of the job involves interpreting biological and biomedical language in order to obtain a meaningful explanation of all living things’ complicated nature. Another challenge lies in the need to search through a large number of articles to find an explanation for the cause of the disease, particularly in the case of complex diseases. PPI extraction can be interpreted in several different techniques, and many machine learning and deep learning techniques have been implemented. Kernel-based machine learning methods have attained good performance, but they require extensive feature engineering, including lexical and syntactic features [9,10,11,12]. In contrast, the application of Neural Networks (NNs) to learn the semantic features and structure of sentences in order to classify them has been regarded as an effective technique for PPI extractions because they do not require an extensive amount of feature engineering like the kernel-based method does [13]. Previous attempts for extracting sentences with relationships between proteins’ names in text were developed using machine learning (ML) and deep learning (DL) schemes. These attempts achieved the highest level of implementation in the field of PPI extraction from text, with deep learning methods being more accurate and achieving greater performance [14,15,16,17]. The objectives of PPI mining can be illustrated as a binary classification problem to distinguish positive sentences from negative ones. Positive sentences would contain the names of proteins in conjunction with relationship words, while negative sentences would represent the opposite [18,19]. Another technique is the named entity recognition (NER) method [20]. This method relies on features engineering and training on datasets so as to recognize proteins’ names in sentences and relationship words.

In the beginning of developing named entity recognition (NER) models, generative models such as Hidden Markov Models (HMMs) were used. Generative models such as HMM have strict rules of learning that rely on the joint distribution of the data, which results in dependent features [21]. The conditional random field (CRF) method was shown to be an effective approach to be used for those types of tasks, especially when learning from widely distributed data, such as the diversity in writing the contexts in the biomedical literature [22]. Discriminative models such as CRF rely on conditional functions with neighboring contextual consideration. This results in more efficient learning when learning from widely distributed data [23]. The pattern-based approach, one of the NLP methods, is the prevalent strategy for building PPI networks. The pattern-based method combines precision and complexity in its pattern design [24,25], but this method requires that the extracted words or sentences match the selected patterns [11]. Although the pattern-based approach has limitations, the high precision of the outturns makes it useful in discovering PPI relation words.

This study proposed a comprehensive method for generating graph figures of PPI networks from only the biomedical literature obtained from the PubMed database, combining the three approaches mentioned above. This method is comprised of three main phases including the development of DL models and the application of patterns to extract information from the text and transfer it to a knowledge graph. In the first step, PPI sentences that only contain the two proteins’ names and their relationship words are extracted from the biomedical literature using a DL with a recurrent neural network (RNN) methodology that competes with the current state-of-the-art methods in extracting PPI from the biomedical literature. The method takes advantage of the AIMed and BioInfer corpora (both available at http://corpora.informatik.hu-berlin.de, accessed on 15 March 2019) [12], which are commonly used reference corpora for PPI extraction applications, to train the DL model. Furthermore, to expand the learning of semantic and syntactic features of the text, a pretrained word embedding vector on more than 20 million biomedical documents from PubMed and more than four billion words of biomedical terms was used to train the DL model [26]. The second step involves developing a named entity recognition (NER) model to label the protein names in sentences using the conditional random field (CRF) method. This model output should be a tagging tool designed to find the protein names in the sentences. Lastly, by using the shortest dependency path model offered by the spaCy library in Python [27], the third step consists of creating the patterns that will be used to extract relationship words from PPI sentences. The final model assigns the dependency parsing labels for the words in the sentences and finds the shortest route between the names of the proteins in the sentences. The starting point of this route usually represents the relation words between the two proteins’ names, sometimes including other dependency labels. The patterns we created to extract the relation words would be defined according to the dependency labels in the shortest path between the proteins’ names in the sentences.

Despite the methods that have been developed previously, either using Kernel-based machine learning or deep learning approaches, most of them require identifying the lexical and syntactic features of words, characters in the words, and sentences and manipulating these features to improve the performance of machine learning or deep learning models. For example, in article [20], the researcher used character embedding for each word in the embedding vector and presented it to the LSTM layer forward and backward to overcome some scientific words such as hyperbilirubinemia and oligonucleotide to be out of the vocabulary. As another example, all previous methods were training their models on AIMed or BioInfer separately, and this might be the reason for the decreased F1 score performance of their models. Furthermore, some studies used both convolution neural networks (CNNs) in conjugation with recurrent neural networks (RNNs) to develop a model with high accuracy, but this would create a model with a complicated structure and take time in the training process. CNN exhibits a hierarchical structure, but RNN exhibits a sequential structure and exhibits better performance when applied to models using textual context [26,28]. The simplest model created from all previous methods is the model in the study [13]. The model is composed of an embedding layer trained on an old biomedical vector developed in 2016 by [29] and one layer of BiLSTM, a recurrent neural network. Their model performance was considerably good.

The methods presented in the current study overcame these problems and simplified the approach by using BioWordVic, the newly developed 2019 word-embedding vector of 4 billion tokens [26], and three-layer BiLSTM neural network in designing the first model in order to achieve better accuracy than the previous models. Also, during training, the first and second models, combining the AIMed and the BioInfer datasets in one dataset, achieved an effective training dataset. Moreover, some previous approaches developed NER models to search for the proteins’ names in the text. In contrast, one objective of the current study is to construct an accelerated method instead of relying only on the NER model alone to identify protein names within an entire abstract or a collection of abstracts. The proposed method involves utilizing the first model to extract sentences related to PPI while discarding other sentences. Subsequently, our NER model will exclusively search for proteins’ names within the extracted PPI sentences. Furthermore, the prior approaches focused on method development rather than constructing a visible protein–protein interaction (PPI) network. Our method is unique in testing it on more than 6000 abstracts and subsequently publishing a protein–protein interaction (PPI) network derived from our method.

The remaining sections are grouped as follows: The Section 2 outlines the new system and explains how the models were created and which methods were chosen in each step of the models’ creation. Then, Section 3 describes the evaluation of the performance of our system consisting of the sentence classification model, the named entity recognition model, and the patterns chosen to drive the PPI network. A thorough review of the current work and related literature is found in Section 4. In Section 5, the impact of the work is summarized to give an insight into the value of using the system created in this study. The PPI system developed in this study depends on extracting the interaction between the proteins from biomedical literature, which means that it would always present updated information in the field of protein–protein interaction networks.

## 2. Materials and Methods

**Figure 1:**
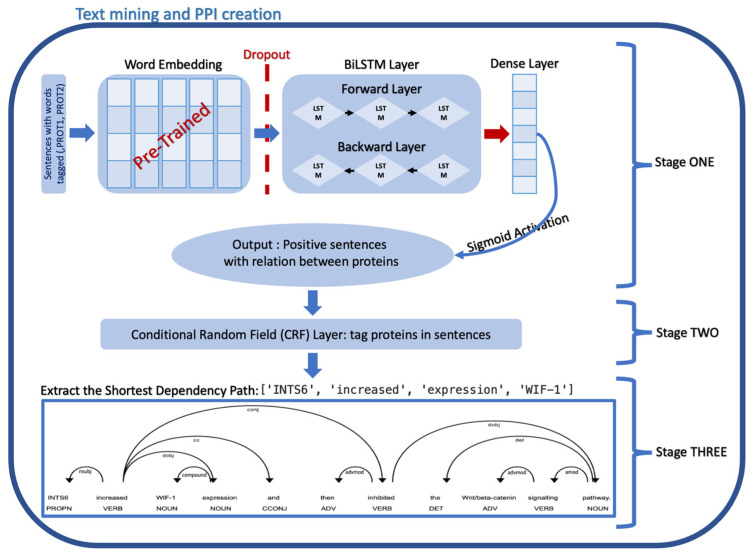
An illustration of the organized steps in creating PPI using machine learning methods in text mining.

The development of the PPI network, which includes extracting the relationship between the proteins from biomedical literature and transferring it to a knowledge graph, was accomplished using machine learning and deep learning methods. Three phases were involved in the process of establishing the PPI network, including developing a sentence classification model, named entity recognition model using a conditional random field (CRF) method, and employing patterns to extract information from the text and transfer it to a knowledge graph (Figure 1).

### 2.1. Text Pre-Processing

The model was trained with AImed and Bioinfer corpus data. The two datasets were integrated and prepped for processing via the Python NLTK library [30]. Combined, the two datasets had approximately 1060 abstracts and 3067 sentences. We performed two types of data processing; however, multiword tokenization was used to ensure that protein names were comprehended in their entirety. Multiword tokenization, for instance, tokenizes Beta and catenin with a dash sign between them if the term beta-catenin is written in this shape (Beta-catenin). The processing of data for the sentence classification model included the terms “PROT1” and “PROT2”. These terms are meant to replace the first and second proteins in the sentences, respectively. When a sentence contains the names of two proteins with a relationship between them, it is considered a positive sentence and is labeled 1 and 0 otherwise. On the other hand, the data processing for the named entity recognition was different. The words in the sentences of each corpus were tokenized, position-tagged, and labeled. The P label was applied to the proteins mentioned in the text, whereas the O label was applied to everything else.

### 2.2. Sentence Classification Model

The model was created in order to distinguish between sentences containing protein relationships and sentences containing no protein links in biomedical abstracts.

#### 2.2.1. Word Embedding

Word embedding is a representation learning technique comprising aligning words with similar meanings and convergingly representing them in a low-dimensional vector space. In the dataset, each word is represented as a vector of positive real values. Specifically, the publicly available pretrained word embedding BioWordVic [26] and GloVe [31] were utilized in this model, with embedding representations of 4 billion tokens and 200-dimensional word embeddings, and 6 billion tokens and 200-dimensional word embeddings, respectively. When using Keras, these pretrained word embedding models were used to create a weight matrix for the embedding layer. Pretrained word embedding, as opposed to one-hot encoding [29], which turns the words into binary vectors, reduces the distance between words with the same meaning and vectorizes them in real numbers. By minimizing the gap between the words, this strategy increases the coverage of words and makes it simpler to recognize the sentences containing information about the protein–protein interaction. On the other hand, one-hot encoding encodes two words with the same meaning in different real numbers. For example, the words (rise and increase) can be synonyms but have a different real number and are not clustered together.

#### 2.2.2. BiLSTM Layer

**Figure 2:**
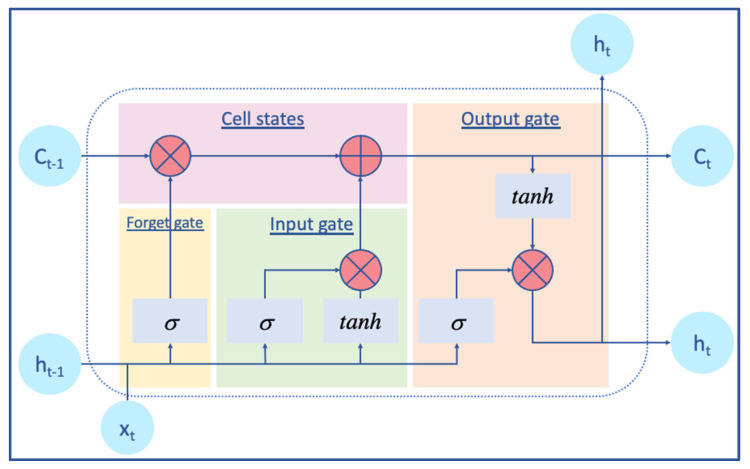
Schematic representation of long short-term memory, LSTM cell, a type of (RNN) that could learn long-term dependencies. Each time iteration t the cell has layer input x and layer output h in light blue circles. The C is the input and the out-of-cell state. The red circles contain arithmetic operations: multiplication and addition. The light blue squares are the gate activation function sigmoid, and tanh is the hyperbolic tangent function. The three gates represented in colors with the cell states control the learning route of the models.

Long short-term nemory (LSTM) artificial recurrent neural network (RNN) is useful in reducing the vanishing gradient mistakes and capturing the semantic information in long sentences because it is fast and efficient [32]. Each LSTM cell has three gates: input gate, forget gate, and output gate. During each time step, the quantity of information that travels through the neurons is controlled by the three gates (Figure 2). Forget gates are used to determine which of the previously hidden states should be reserved. Specifically, the forget gate enables the LSTM cell to be effective and scalable for a wide variety of sequential data feature learning. The input gate decides which of the currently hidden states should be retained. The cell state updates the cell states from the forget gate, output gate, and input gate. The output gate decides the next hidden state [33,34].

Each LSTM cell’s mathematical representation and the equations governing its three gates are as follows:

*i_t_* = *σ* (*W_ix_ x_t_* + *W_ih_ h_t_*_−1_ + *b_i_*) (1)

*f_t_* = *σ* (*W_fx_ x_t_* + *W_fh_ h_t_*_−1_ + *b_f_*) (2)

*o_t_* = *σ* (*W_ox_* + *W_oh_ h_t_*_−1_ + *b_o_*) (3)

*c_t_* = *f_t_* × *c_t_*_−1_ + *i_t_* × *tanh* (*W_cx_ x_t_* + *W_ch_ h_t_*_−1_ + *b_c_*) (4)

*h_t_* = *o_t_* × *tanh* (*c_t_*) (5)

where *i* is the input gate, *f* is the forget gate, *o* is the output gate, *c* is the cell states, *x_t_* is the word embedding vector, *h_t_*_−1_ is the hidden state, the *W*’s are the weight matrices, The *b’s* are the bias vectors, *σ* is the sigmoid function, and tanh is the hyperbolic tangent function.

BiLSTM is well suited for use in sentiment analysis and text classification models. The LSTM cells in the forward and backward layers of the recurrent neural network form the structure of the BiLSTM recurrent neural network. To obtain optimal performance, it is important to train on both the input sequence and its reverse duplicate. As a result, the sequence categorization classification is swiftly and thoroughly acquired.

**Figure 3:**
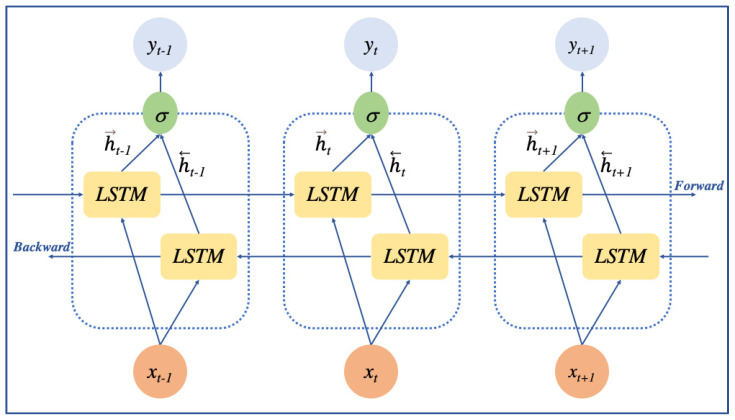
Schematic representation of bidirectional long short-term memory (BiLSTM) network. The output of the word embedding layer is taken as input x. The BiLSTM network will train both the original sequence and its reversed counterpart. The sigmoid function will aggregate the result of both training directions and represent it as an output y.

As demonstrated in (Figure 3), *x_t_* is the word embedding vector. $\vec{h}\;$ is the forward hidden layer, $\overset{\leftarrow }{h}$ is the backward hidden layer, and *y_t_* is the joining outputs from the forward and backward hidden layers. The output layer values are processed as follows:

(6) h→t=σ (W →x hxt+W→h hh→t−1 xt+b→h)

(7) h←t=σ (W←x hxt+W←h h h←t+1 xt+b←h)

(8) $$y_{t}=W\underset{h\;y}{\to }{\vec{h}}_{t}+W\underset{h\;y}{\leftarrow }{\overset{\leftarrow }{h}}_{t}+b_{y}$$

where *W* is weights matrices, *b* is the bias term, *σ* is sigmoid function, and *h_t_* is the hidden state. A dense layer was added at the end to ensure that all of the output neurons in the BiLSTM neural network were fully connected. Because of the usage of two classes, positive and negative sentences, the output prediction of the model with a Softmax activation function is performed using the dense layer. This layer predicts a multinomial probability distribution. In this case, the prediction probability range is 0 to 1. A prediction of less than 0.5 is regarded as a negative prediction, whereas a prediction of equal to or greater than 0.5 is seen as a positive prediction.

### 2.3. Named Entity Recognition Model Using Conditional Random Field

The model has been developed to tag the names of proteins after they have been extracted from positive sentences that describe the relationships between proteins.

#### Entity Tagging

After text pre-processing for the two datasets (Aimed/Bioinfer) and using the letters (P) and (O) to label the protein names and other words, respectively, the dataset is used to train the model using the sklearn-CRFSuite library in Python (version 3.8) [35]. Conditional random field (CRF) is a statistical probabilistic modelling method used for structured prediction. Because it is only two labels, the NER-CRF model would perform better than utilizing neural network (NN) models [36]. The output of the trained model is a tagging tool to search and recognize the protein names in the sentences.

### 2.4. Relation Extraction

**Figure 4:**
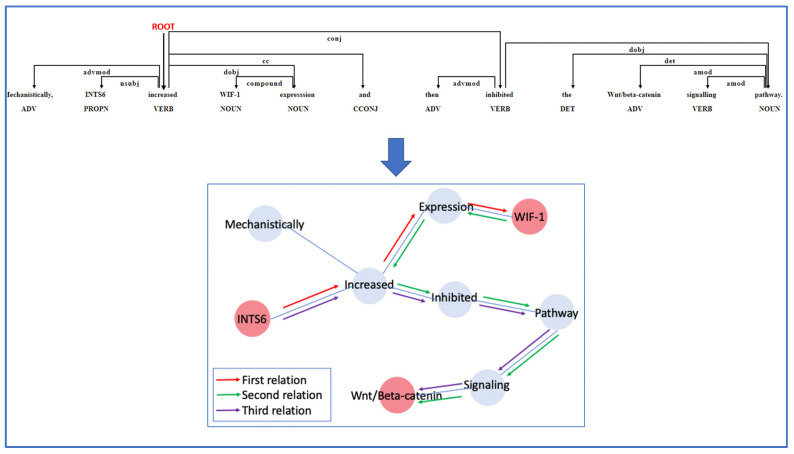
The shortest dependency path of the sentence “Mechanistically INTS6 increased WIF-1 expression and then inhibited the Wnt/beta-catenin signaling pathway” and the relation extraction exemplification.

Following the selection of sentences containing relationships between proteins using the sentence classification model and the tagging of proteins names using the NER-CRF model, the shortest dependency path model in spaCy was then employed to extract the shortest path between the names of the proteins in the selected positive sentences. Interaction sentences in PPI are composed of nouns and verbs. The verbs are almost always the focal point of all sentences. Dependency parsing illustrates the sentences as trees and recognizes and labels the center of the sentences as the ROOT of the tree, which is usually reflected in the verbs. The dependency labels for the remaining words are assigned by the spaCy shortest dependency path model based on the syntactic structure of the sentence (Figure 4).

It is necessary to transform each sentence’s syntactic structure into a graph representation, which is performed using the networkx module in Python [37], in order to find the shortest path between two protein names. For example, the sentence “Mechanistically INTS6 increased WIF-1 expression and then inhibited the Wnt/beta-catenin signaling pathway” has three tagged protein names: INTS6, WIF1, and Wnt/beta-catenin. There are a definite number of relations that can be extracted based on the number of protein names present, and in the example given in the preceding line, there are three relations that can be extracted (Figure 4). Having collected the phrases with the shortest dependency paths, the dependency labels were examined and investigated in order to identify a common pattern that could be used to extract the accurate relationship. The first protein name dependency label is usually subj, and the second protein name dependency label is usually obj. Discovering the range of dependency labels in which the relation words should be placed was the difficult part of the task. Usually, the ROOT dependency label (the verb in the middle of the sentence) defines the relation word, but in some shortest dependency path sentences, the ROOT dependency label in conjugation with the amod or dep dependency label is found to explain the relation words even more clearly. Other dependency labels were discovered to describe the relationships in the sentences, and these were taken into consideration as well. The pattern was defined, and the relationship was extracted using the spaCy shortest dependency path model and matcher library.

## 3. Results

The approach developed in this paper to extract PPI networks from biomedical publications involves text dataset preparation, sentence classification model generation, named entity recognition model creation, and relation extraction. In Section 3, each of the preceding approaches and the features employed in each method, as well as their impact on the models’ performance, are described. In addition, the performance of the methods developed in this study is compared with other previous state-of-the-art approaches to demonstrate one of the strengths of the proposed model.

### 3.1. Data Preparation

Two datasets were prepared to train the NLP models. For the sentence’s classification model, AImed and Bioinfer corpora were used and processed. The processing includes joining all of the sentences from the two corpora, multiword tokenization, and changing the protein names in sentences, as well as labelling positive sentences with 1 and negative sentences with 0. The number of positive (sentences contain relationships between the proteins) and negative (sentences do not contain relationships between the proteins) in the AIMed/BioInfer PPI dataset are 3067 total sentences, 1267 total positive sentences, and 1800 total negative sentences.

For the named entity recognition model, AImed and Bioinfer corpora were also used and processed, but we only consider the positive sentences that exhibit a relationship between proteins. Processing the sentences included multiword tokenization, position-tagging, and labelling the words in the sentences. Label P was considered for the proteins’ words and O for other words. So, the datasets were transformed to a dataset with words labeled as P or O. It is estimated that there were 31,472 words with an O label and 4078 words with a P label.

### 3.2. Sentence Classification Models

Sentence classification is one of the natural language processing (NLP) tasks that labels sentences according to predefined classes. In the context of this study, the primary sentence classes are positive and negative, with positive sentences indicating the presence of two protein names and a relation between them. We followed the architecture of the previous state-of-the-art methods devised in the field of PPI extraction, which consists of a neural network layer followed by a pretrained word embedding layer. The following describes the initialization of the two layers:

#### 3.2.1. Word Embedding Initialization

We utilized the BioWordVic and GloVe models of vector representations for words while modelling the sentence classification model. BioWordVic was trained on 28 million PubMed articles and 2 million MIMIC III Clinical notes. GloVe was trained using news stories and the Wikipedia database. Because various earlier state-of-the-art models have been created utilizing these two pretrained word embedding models, we aimed to compare and contrast the differences and precisions of the models developed in this paper using those pretrained word embedding techniques.

#### 3.2.2. The RNN Layer

We constructed four sentence classification models using Keras v 2.13.1 with TensorFlow v 2.13 (Chollet and others). In the first two models, input sentences from both datasets (AIMed and BioInfer) are fed into the model, and the words in the input sentences are given their real number representation through the word embedding layer, which contains the weight matrix of the above-mentioned pretrained word embedding vectors, before being transferred to the neural nodes. We set the number of BiLSTM nodes to 100, the dropout rate to 0.5, and the learning rate for the Adam optimizer to 1 × 10^−4^. In the second two models, we stacked three hidden BiLSTM layers after the word embedding layer that has a weight matrix of the two pretrained word embedding models. Also, we dropped the number of nodes in all BiLSTM layers to 32. There is no predetermined number of nodes used for the BiLSTM layer. The number of nodes needs to be nearly compatible with the number of the input sequence of 120. In the model that has one BiLSTM layer, 100 nodes were used, and in the model that has three hidden BiLSTM layers, 32 nodes were used. The total number of nodes in the three hidden layers is 96 (32 × 3); that is, the input sequence passes 96 nodes forward and 96 nodes backward. Although our network would be considered small, when many dropout rates were tested, the best rate was 0.5 for the dropout layer and 0.2 for the recurrent dropout layer. The activation function is SoftMax because the model has two labels: 0 for negative sentences with no PPI and 1 for positive sentences with PPI. Adam optimization is a method for stochastic gradient descent that utilizes adaptive methods to estimate first-order and second-order variables. Rates from 0.01 to 0.0001 were tested with the finding that the lower the rate of learning, the higher the accuracy of the model prediction. The following are the parameters that govern the sentence classification models are shown in (Table 1).

#### 3.2.3. Measures of Performance

The F1 score, which is often used to measure the performance of classification models, was utilized to evaluate the models. The F1 score is determined by the precision and recall scores that describe the model’s performance. The final two scores depend on the model’s true positive, true negative, false positive, and false negative predictions. The following equations define the precision, recall, and F1 score:

(9) $$Precision=\frac{TP}{TP+FP}$$

(10) $$Recall=\frac{TP}{TP+FN}$$

(11) $${F1}_{Score}=2\times \frac{Precision\times Recall}{Precision+Recall}$$

We utilized the classification report matric in the Python sklearn library. The data was divided into 60% training data and 40% testing data. The global average F-score was calculated in order to evaluate the overall performance of the models regardless of the class.

### 3.3. Named Entity Recognition Model Initialization

**Figure 5:**
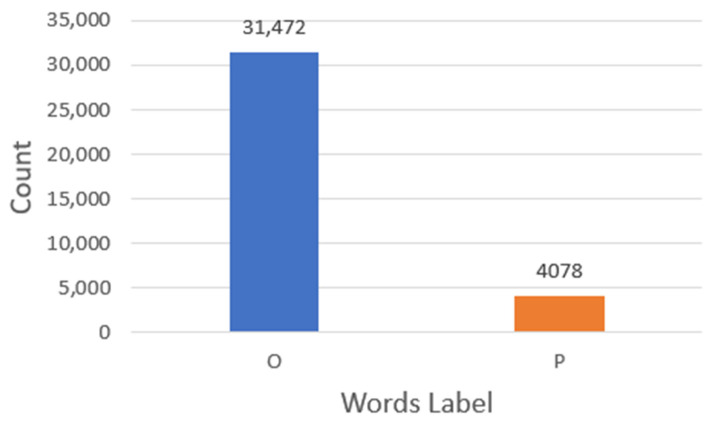
Statistics for the NER model dataset. P label for protein names and O label for other words.

In this stage, the Python sklearn-CRFSuite module was utilized. Initially, the training data consisted of a list of each word, its position tag, and its label (P or O). This data list would be utilized to train the NER model. Certain conditions are implemented while training the model, such as defining the surrounding word features. Finally, a labeler that recognizes protein names in sentences will be created. When evaluating the model’s efficacy, the weighted F1 score was employed due to the presence of label imbalance in the dataset, as there were only 4078 words with portion label (P) and 31,472 other words with label (O) (Figure 5). The metrics for each label are calculated, and their average weight by support is determined using the weighted F1 score, how many actual occurrences there are for each label. Table 2 shows the parameters that were used in the NER-CRF model.

### 3.4. Relation Extraction Implementation

The shortest dependency path model provided and modelled by the spaCy library in Python was used to extract the shortest path between the proteins in the PPI sentences after using the first two proposed models to first extract the sentences that have PPI and then extract and label the protein names in the PPI sentences. This model’s unique contribution is the way it assigns the dependency labels for words according to the norms of sentence formation. We found that words with a ROOT dependency label locate and define the relationship between the two protein names in the sentences. The dependency label range of the first protein is usually (‘nsubj’, ‘amod’, ‘compound’), and the dependency label range of the second protein is usually (‘dobj’, ‘pobj’, ‘npadvmod’, ‘appos’). The patterns we chose to locate and define the relationship words are as follows: (‘DEP’: ‘amod’, ‘OP’: “*”), (‘DEP’: ‘conj’, ‘OP’: “*”), (‘DEP’: ‘ROOT’, ‘OP’: “*”), (‘DEP’: ‘acomp’, ‘OP’: “*”), and we used matcher library in Python to locate the relationship words from the sentences, with their dependency labels matching the arrangement we created in the patterns.

### 3.5. Evaluation of Models’ Performance

The aim of this section is to elucidate how the learning performance of the models developed in this study was determined. Also, the sequence of using these models in the order of searching for positive sentences containing the relation between proteins, followed by searching for the names of the proteins and the relation words, was successful when compared to a manual method of reviewing abstracts related to seven proteins.

#### 3.5.1. Sentence Classification Models

Lately, BiLSTM neural networks have been shown to be effective in PPI tasks. With the differences in text pre-processing (e.g., joining the two datasets of AIMed/BioInfer, and multiword tokenization to fully obtain the protein names), the hyperparameter settings, and the models used as pretrained word embedding, we are comparing our four created models with state-of-the-art previously created models that are nearly similar to the structure of our models. The following table shows the performance of the four models compared to other models. Because the previous models were trained on Aimed and BioInfer separately, we took the average F1 scores for both models (Table 3).

Taking advantage of the pretrained word technique and the availability of its models, the BioWordVic model performed better than the GloVe model between our models (3 and 4), (5 and 6). This means that the RNN had the ability to automatically collect contextual information from the BioWordVic model more than the GloVe model in terms of PPI tasks. If we compare our models 3 and 4 to 1 and 2, our models nearly achieved similar accuracy to the previously created models in the case of 1 and 2, with differences in the word embedding vectors that the models were trained on. Stacking the three hidden layers of BiLSTM in our models 5 and 6 further improves the models’ performance and increases their accuracy.

#### 3.5.2. NER-CRF Model

For named entity recognition (NER) models and capturing biological molecules (e.g., DNA, RNA, Protein) from texts and biomedical abstracts, CRF models proved to be effective in the field. Our NER-CRF model trained on 35,550 AIMed/BioInfer corpus words achieved an F1 score of 0.98. Previous NER attempts developed tools using the CRF method. ABNER is a biomedical NER tool used to tag biomedical entities in texts using CRF. They trained the model on an NLPBA corpus (modified version of the GENIA corpus) that has 22,402 training and testing sentences and a BioCreative corpus that has 10,000 training and testing sentences. The F1 scores of the tagging models are 0.705 on the NLPBA corpus and 0.699 on the BioCreative corpus [38]. ProNER is another named entity recognition model that recognizes the protein names in sentences using the Bayesian probability-based Finite State Machine (BFSM). BFSM is based on the conditional probability of identifying specific entities in texts. The model was trained on the GENIA corpus with F1 scores of 0.907 [16]. The differences between our model and the previous model lie in the use of different corpus, number of sentences and the words it was trained on, and also in that we developed our model to be trained on two types of labels (O and P).

### 3.6. Testing the Models and PPI Network Creation

Four patients with Autism Spectrum Disorder (ASD), as mentioned in study [39], with a confirmation of the genetic variants implicated in ASD, are described in Table 4.

After an exhaustive manual annotation of the protein databases and literature curations, a streamline of the interactions between these proteins and the other molecules in the Wnt and mTOR pathways was discovered, and convergence was found between these two signaling pathways according to the evidence extracted from previous studies (Figure 1). The manual curation included the following: (i) defining the positions where the genetic variants are located, (ii) identifying the function of the genetic variants position, and (iii) searching and reviewing previous studies for the proteins (Table 4), including finding information about their paralogs and protein families. The main databases used for the manual curation were UniProt, the conserved domain database (CDD), PROSITE, iPTMnet, and PubMed. For example, INTS6L is paralog to INTS6. A review of the protein’s profile in UniProt and conserved domain databases confirms the comprising of the von Willebrand factor type A (VWFA) domain in the N-terminus, a DEAD-box motif, and a functional C-terminal domain. The mutation of INTS6L is not located in any of the previous critical functional locations. Also, the protein was found to be not well studied when compared to the number of studies extracted from PubMed, but *INTS6*, its paralog, has been well studied in previous studies; the latter protein was discovered to be a critical element affecting restrictive dorsal cell growth through the Wnt pathway [40]. Similar reviews were undertaken for the other proteins in the protein databases and PubMed to see if their mutations are positioned in critical function locations on the proteins or not.

**Figure 6:**
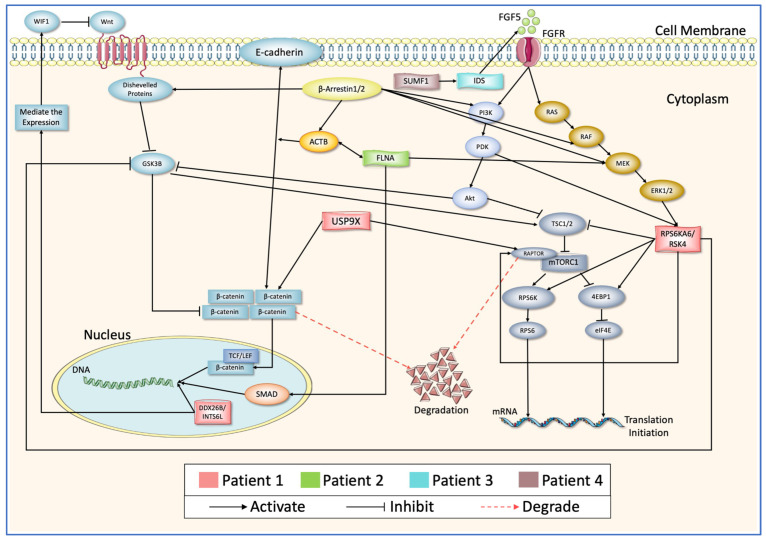
Schematic representation of the mutated proteins in four patients colored with red, green, cyan, and brown with arrangements of their roles on Wnt and mTOR signaling pathways.

From the summary of the interactions between the proteins (Figure 6), it can be concluded that INTS6, the paralog of INTS6L, has an important effect on the Wnt pathway, and its mutation is connected to dorsal cell growth. It increases the expression of WIF-1, the inhibitor of Wnt protein, and regulates the activity of the Wnt pathway [40,41,42]. USP9X physically interacts with RAPTOR and β-catenin to prevent their degradation through the proteasomal degradation pathway [43,44,45]. RPS6KA6/RSK4 is abundantly expressed in the brain, and its mutation is associated with non-specific mental retardation and development defects [46,47]. RSK enzymes phosphorylate RAPTOR to activate the mTOR pathway [48,49,50]. Also, they inhibit the enzyme activity of GSK3β, leading to the accumulation of β-catenin and cell survival [51]. FGF5 binds to FGFR1 receptors to activate different pathways, including PI3K/Akt, RAS/MAPKs, and PLCγ/DAG. These pathways are engaged in a wide range of cell proliferative processes, including embryonic growth, cell growth and survival, and tissue repair [52]. Altering signaling has been tied to many diseases, such as bone diseases, cancers, dwarfism, hair growth, neural plasticity, and ASD [53,54,55]. The FLNA actin-binding mechanism is vital for cell adhesion processes. There are 24 repeat areas in the protein’s C-terminal region, and these regions are involved in protein interactions. *FLNA* mutations alter important cellular processes and have been linked to several disorders, including ASD [56,57]. The mutation in FLNA is located in repeat 22, where the protein interacts with β-Arrestin to activate MEK and SMAD to translocate to the nucleus and transcribe the targeted genes [58,59,60]. *IDS* is a sulfatase lysosomal enzyme important in protein metabolism and specifically in degrading large carbohydrate molecules, glycosaminoglycans, from its substrate dermatan sulfate and heparan sulfate [61]. Heparan sulfate is a specific and central component in FGF/FGFR dimerization and binding [62]. SUMF1 is a cofactor that enhances the activity of sulfatase enzymes, such as *IDS* [63]. Mutation in SUMF1 causes a deficiency of sulfatase enzymes because it is an essential molecule in the post-translation modification of these enzymes and a critical molecule present in the modification of cysteine residue to the formyl glycine residue site [64].

**Figure 7:**
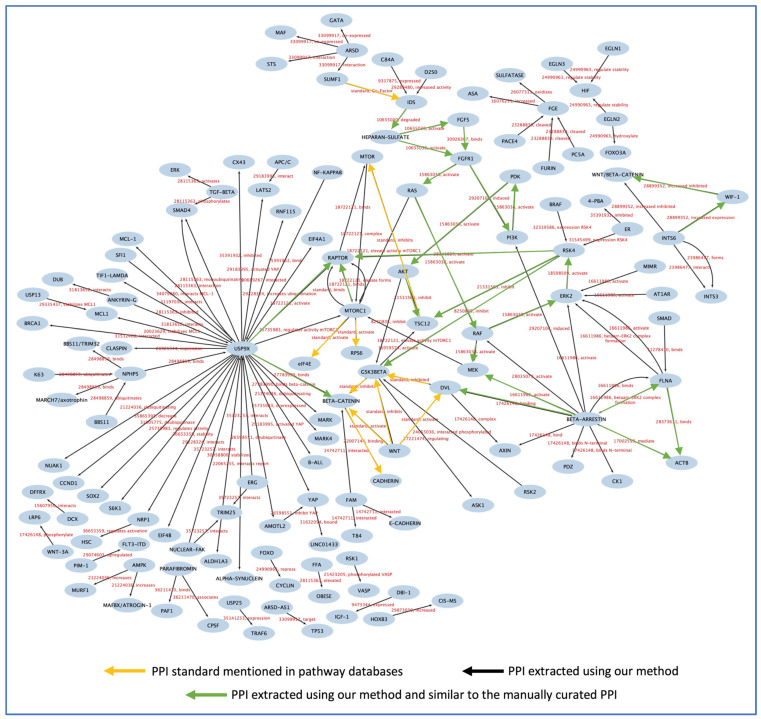
The PPI network was created using our text mining method. The manual curation results in Figure 6 are included in this figure and are represented with green arrows.

Using automated text mining and pattern recognition techniques, a PPI network diagram was generated (Figure 7). The PubMed database was searched for all articles pertaining to USP9X, INTS6L, RSK4, IDS, and SUMF1, and all PMIDs, including those of manually curated articles, FGF5 and FLNA, were collected. There was a total of 6027 articles compiled to generate the PPI figure. The PPI outlined in Figure 7 was more informative regarding the other proteins that interact with USP9X, INTS6L, RSK4, IDS, FGF5, FLNA, and SUMF1. Remarkably, this also demonstrated whether or not the later proteins interact with each other. The interactions found manually between the proteins in Figure 6 have been identified in the PPI network generated through the use of text mining and pattern recognition algorithms. The interaction terms and the PMIDs extracted from the articles were added to the edges so that the original paper describing the interaction between the two proteins’ nodes could be reviewed.

## 4. Discussion

Here, our results demonstrated that the system developed for PPI extraction successfully extracted the manually curated relationships between seven proteins and discovered additional relationships. The majority of PPI extraction research focuses on improving the accuracy of their models rather than validating their models to demonstrate actual results of PPI network creation and compete with existing tools that generate PPI networks. Testing our designed system was the most crucial phase in proving our theory regarding the formation of PPI networks. We have already manually evaluated 25 articles for seven proteins identified as the cause of autism in four patients. To evaluate our methodology, we extracted all abstracts pertinent to the seven proteins, totaling 6027, from the scientific literature. After applying our system to the abstracts, we generated an expandable image of all the proteins that interact with the seven proteins. What was intriguing was the inclusion of the manually curated PPI network (Figure 6) in the expandable image.

In this study, we proposed developing and combining effective mining and generation techniques for PPI networks. In addition, we organized these methods in a manner that helps facilitate the development of PPI networks. When we first created the sentence classification model, we were aware that the application of deep learning has significantly supported PPI initiatives in recent years. The effect of deep learning models is determined by their architecture. In general, the arrangement of the word embedding layer and the neural network layer was the most commonly used arrangement. The word embedding techniques can influence the classifier’s performance. To accelerate feature learning, the semantic and syntactic features of incoming text are embedded in their distribution representations. In our project, using the BioWordVic, the most recently developed word vector model for biomedical texts and which has never been used in any previous study, improved the deep learning model’s feature learning more than using the CloVe word embedding model, which is trained on news and Wikipedia articles. Then, our sentence classification model demonstrated that stacking three hidden BiLSTM layers as opposed to utilizing a single BiLSTM layer improved the efficacy of extracting sentences with PPI. Moreover, our model showed an improvement of 21% and 13% in precision compared to the previous studies [13,17], respectively.

After successfully extracting the sentence with PPI, we needed a model that automatically searched for protein names in these sentences. NER was the most suitable strategy for this objective. The CRF method performed remarkably well in identifying protein names in the biomedical abstracts, as it was the most dependable and effective method we evaluated. Our identifiers for protein words in the AImed/BioInfr corpus sentences have been simplified. We labeled non-protein words with the letter O and protein words with the letter P. Previous methods included P1 and P2 labels for the names of the two proteins because they relied on NER models to find PPI sentences [16,38]. In our case, we relied on the sentence classification model to obtain all PPI sentences, and then we required an automated method to extract the protein names from these sentences in order to determine their relationship. Because of this, our model has a superior F1 score of 0.97. Incorporating the words that define the relationship between the proteins was challenging. We were compelled to utilize the shortest dependency path model offered by spaCy, the Python library for advanced natural language processing (NLP). This model supplies dependency identifiers for every word in the sentence. These identifiers represent the semantic characteristics of words, making it easier to locate related words in sentences. Then, we defined and implemented patterns containing dependency labels. This method, which had never been used before, was exceedingly effective at deriving relational words between protein names in sentences.

Only our method, which consists of two NLP models and a pattern recognition technique, shown in Figure 1, was successful in extracting the PPI and generating the PPI network. Prior to developing our method, we experimented with various NLP techniques, such as locating protein entities in the text and then extracting PPI, but this would increase the number of sentences the model must search for and slow down the process. Our method was the only reliable means to perceive and extract the PPI network. When comparing the online PPI tools STRING and GENEMANIA to our method, which were used as they are the only tools available that are comparable to ours as both use text mining and NLP techniques, we found that our method is more accurate in detecting PPI than STRING or GENEMANIA. We tested the sentence “Our pipeline uncovered variants in 15 ASD-candidate genes, including 5 (GLT8D1, HTATSF1, OR6C65, ITIH6 and DDX26B) that have not been reported in any human condition.” using our sentiment analysis model, STRING, and GENEMANIA. However, we still have some challenges to address, such as the problem of searching through a large number of abstracts and the availability of full texts, which continue to be obstacles to the implementation of our system, as in order to successfully complete the task, it is necessary to have access to high-performance computers and sufficient storage capacity. Of note, our methodology involves inputting specific protein entries and retrieving the PubMed Identifiers (PMIDs) associated with research related to the given protein names from the PubMed database. Subsequently, we extracted the abstracts of these PMIDs and proceeded with the extraction of the protein–protein interaction (PPI) network. The current algorithm does not encompass synonyms associated with the protein, and this step is left for future work. Furthermore, in the future, it is essential to provide users with the option to upload either a complete article text or supplementary table for the purpose of extracting the PPI network. Also, a hypothesized PPI or PPI driver achieved via a computational method is considered a bias. The limitations of digitizing tasks appear in these situations. Once an article is published and the PPI is cited, computational learning methods are unable to distinguish between computer- and laboratory-based work.

However, the field of NLP is swiftly evolving. For the upkeep of our method, the NLP model must be revised continuously to remain current in relation to developments in the field of NLP. As mentioned earlier, BiLSTM RNN was chosen over CNN because the developed models trained on textual datasets and RNN have a sequential architecture more suitable for a sequence of words. GRU is another form of RNN. Because of their shared architecture and similar performance characteristics, LSTM and GRU are often compared as two variants of the same model. LSTM has three gates in its cells: the input gate, the output gate, and the forget gate. The GRU architecture incorporates two different gates within its cells, namely the update gate and the reset gate. One notable characteristic of these gates is their ability to retain previous data without eliminating them, even if they become irrelevant to the prediction. In LSTM, the forget gate is used to selectively keep or discard specific components of the preceding cell state. The GRU update and reset gate is better in keeping the data and would be more beneficial in generative AI models [64]. In our case, the analysis was restricted to a dataset containing PPI sentences, and using either of the models is a suitable approach for training our model. When mentioning generative AI, BioGPT is the most recent innovation in the field of biomedical NLP, and its development continues. Currently, BioGPT can be utilized to seek relevant materials. If you input the term KRAS, for example, a list of sentences that mention KRAS in the biomedical literature will appear. This model has the ability to expedite the extraction of sentences from an abstract or full-text article that mentions the target protein. This model lacks information about the article from which the sentences were extracted, which is a disadvantage. Additionally, it is not adaptable to the requirements of all consumers. In other words, the model requires coding expertise and is not an application. This model can only be utilized by bioinformaticians and software developers in the biomedical field. Therefore, the use of this model is questionable in the field of text mining when generating a PPI network.

## 5. Conclusions

In this study, an automated method for defining and constructing a protein–protein interaction (PPI) network was described. This paper summarizes the use of artificial intelligence (AI) approaches in text mining, which provides the opportunity to learn more about the other factors that contribute to the development of a disease. This strategy has the potential to reduce the amount of time spent manually searching for reliable information and to streamline the development of a comprehensive picture of the events leading to the onset of disease phenotypes. Although we focused our research on genetic variants associated with ASD, we have found that this strategy may be applicable to other types of disorders and diseases. As part of our future research, we are discussing the creation of a web tool that is accessible to the scientific community, with the expectation that additional discoveries will be made in the future. In addition, a generative AI method should be implemented so that our model can retrain itself without human intervention in the process of updating the system.

## Figures and Tables

**Table 1:** Sentence classification model parameters.

Parameter	Combination of AIMed/BioInfer
Maximum of sentences length	120
BiLSTM units	100
Hidden BiLSTM units	32
Dropout rate	0.5
Recurrent dropout rate	0.2
Optimization algorithm	Adam
Activation function	Softmax
Learning rate	1 × 10^−4^
Epochs	40
Batch size	128

**Table 2:** Conditional random field (CRF) model parameters.

Parameter	Combination of AIMed/BioInfer
Number of words	35,550
Algorithm	lbfgs
C1	0.1
C2	0.1
Maximum iterations	100
All possible transitions	False

**Table 3:** Comparison between all the models created in this project (3, 4, 5, and 6) to similar previous models created before (1, 2). Class (1) means sentence labeled with 1, and class (0) means sentences labeled with 0.

Method	Positive Class (1) F1 Score	Negative Class (0) F1 Score	Cumulative F1 Score
Bidirectional LSTM + CNN + word embedding (BioNLP) + SDPs embedding [17]	-	-	0.74
Bidirectional LSTM + word embedding (BioNLP) [13]	-	-	0.82
CloVe pretrained word embedding + BiLSTM layer	0.73	0.75	0.74
BioWordVic pretrained word embedding + BiLSTM layer	0.76	0.77	0.77
CloVe pretrained word embedding + 3 hidden layers of BiLSTM	0.94	0.92	0.93
BioWordVic pretrained word embedding + 3 hidden layers of BiLSTM	0.94	0.95	0.95

**Table 4:** The number of autism patients and the genetic variants considered in this article and collected from the study of [39]. DC: disease-causing; PD: possibly damaging; B: benign; D: damaging.

	Gender	Clinical Demographic Information	Protein Name	Variant Position	Effect of the Variant	
					Mutation Taster	PolyPhen
Patient 1	F	Language delay and regression	DDX26B/INTS6L	p:E435V	DC	PD/0.843
			USP9X	p:Y1268C	DC	B/0.007
			RPS6KA6/RSK4	p:Q512R	DC	B/0.195
Patient 2	M	NR	FGF5	p:S84L	DC	D/1.0
			FLNA	p:Y2360A	DC	D/0.971
Patient 3	M	Language delay	IDS	p:D175E	DC	PD/0.94
Patient 4	M	Language delay	SUMF1	p:Q237R	DC	D/1.0

## Data Availability

All data generated or analyzed during this study are included in this published article and its supplementary information files (“Al-Mubarak, B.; Abouelhoda, M.; Omar, A.; AlDhalaan, H.; Aldosari, M.; Nester, M.; Alshamrani, H.A.; El-Kalioby, M.; Goljan, E.; Albar, R.; et al. Whole exome sequencing reveals inherited and de novo variants in autism spectrum disorder: a trio study from Saudi families. *Sci. Rep.* **2017**, *7*, 5679. https://doi.org/10.1038/s41598-017-06033-1”) [39]. The PPI model and other code are shared in https://github.com/lnezamuldeen/PPI_creation_models (accessed on 15 October 2023).
